# Antibiofilm Peptides and Peptidomimetics with Focus on Surface Immobilization

**DOI:** 10.3390/biom8020027

**Published:** 2018-05-16

**Authors:** Athina Andrea, Natalia Molchanova, Håvard Jenssen

**Affiliations:** Department of Science and Environment, Roskilde University, Universitetsvej 1, 4000 Roskilde, Denmark; atan@ruc.dk (A.A.); nataliam@ruc.dk (N.M.)

**Keywords:** surface-immobilized peptides, peptidomimetics, antibiofilm

## Abstract

Bacterial biofilms pose a major threat to public health, as they are associated with at least two thirds of all infections. They are highly resilient and render conventional antibiotics inefficient. As a part of the innate immune system, antimicrobial peptides have drawn attention within the last decades, as some of them are able to eradicate biofilms at sub-minimum inhibitory concentration (MIC) levels. However, peptides possess a number of disadvantages, such as susceptibility to proteolytic degradation, pH and/or salinity-dependent activity and loss of activity due to binding to serum proteins. Hence, proteolytically stable peptidomimetics were designed to overcome these drawbacks. This paper summarizes the current peptide and peptidomimetic strategies for combating bacteria-associated biofilm infections, both in respect to soluble and surface-functionalized solutions.

## 1. Introduction

For the past hundred years, most research on pathogenic bacteria has focused on combating acute infections resulting in the development of effective vaccines and antibiotics. Acutely evolving infections are thought to be caused by planktonic “free-swimming” bacteria, and can generally be successfully treated. However, bacteria forming sessile multicellular aggregates called biofilms cause most persistent infections in humans [1,2]. Biofilms are the predominant lifestyle of bacteria and account for at least two thirds of all infections in humans. Bacteria within biofilms are embedded in a self-produced extracellular matrix, a scaffold with vital properties of retaining and protecting the biofilm [3]. This matrix consists of glycopeptides, polysaccharides and a number of biomolecules e.g., proteins, extracellular DNA, and lipids [3]. Bacteria can engage in intercellular cell–cell signaling (also called quorum sensing) to coordinate gene expression [4,5]. This trait allows bacteria to monitor their environment for the presence of other bacteria and to respond to fluctuations in the number and/or species present by altering particular behaviors [6]. The major challenge associated with treatment of biofilms is their increased tolerance to antimicrobial agents [1]. The extracellular matrix can prevent or reduce diffusion of antimicrobial agents as well as bind them [7]. Other factors contributing to biofilm resilience include decreased growth rate, expression of biofilm-related resistance genes, and presence of dormant persister cells, virtually tolerant to all conventional drugs [1,8]. Due to their unique nature, most critical characteristics of biofilm-based infections are extreme resistance to antibiotics and many other conventional antimicrobial agents, as well as an extreme capacity for evading the host defenses. As a result, biofilms are up to 1000 times more resistant to antibiotics compared to their planktonic counterparts; hence, they cause infections that often turn out to be difficult to treat and lead to development of a chronic state [9].

Initiation of a biofilm infection is classically associated with bacterial adherence, either to an abiotic surface or, just as frequently, to a host cell surface, though entrapment of bacteria in e.g., a mucus layer also can result in development of a biofilm infection. Adherence to host tissue can lead to a number of diseases, like chronic wound infection, chronic otitis media, chronic osteomyelitis, chronic rhinosinusitis, recurrent urinary tract infection, endocarditis and cystic fibrosis-associated lung infection [10]. Biofilm-forming bacteria are also associated with chronic inflammatory diseases such as Crohn’s disease as well as dental caries and periodontitis [11,12].

The second type portrays colonization of bacteria on abiotic surfaces such as those of indwelling medical implants. Despite the steady developments in surgical practices and biomaterial design, biomaterial-associated infections account for a large portion of all nosocomial infections and pose an enormous global economic burden, as they can compromise or even cause the failure of the medical implant. In case of infected implants, the best and often only option is removal and replacement, which often require surgical procedures, thus imposing greater costs to healthcare system providers, as well as inconvenience and increased risk for the patient [13]. The surface of medical implants can be colonized by bacteria during the implantation process, or at a later time point [14,15], putting the performance and longevity of the implant at risk. For example, in catheterized patients, catheter-associated urinary tract infections account for roughly a quarter of the infections in intensive care units [16,17].

Conventional approaches, including prophylactic and systemic administration of antibiotics, or even combinations of them, fail to adequately treat biofilm-associated infections. Additionally, none of the antibiotics currently available in the clinic have been specifically designed to eradicate microbial biofilms. For example, despite aggressive and intensive antibiotic therapy, chronic *Pseudomonas aeruginosa* lung infection in cystic fibrosis patients is almost never fully eradicated [18,19]. Similar trends are observed in combating infections on medical implants, due to failure of establishment of a sufficient antibiotic concentration at the implant site [20,21,22]. Hence, a novel therapeutic approach for biofilm eradication as well as localized delivery of antimicrobial agents, which will tackle the problems of systemic drug administration is highly desirable [15,23,24,25].

Therefore, in the wake of the growing antimicrobial resistance and the severe lack of new antibiotics, antimicrobial peptides (AMPs) have emerged as an attractive tool to combat both bacterial biofilms and antibiotic resistant strains [26]. As a part of innate immune system, AMPs exhibit broad-spectrum activity and provide a first line of defense of virtually all organisms [27]. Today, all reported naturally occurring and synthetic AMPs are collected in databases, e.g., Data Repository of Antimicrobial Peptides (DRAMP), which contains more than 4500 sequences of antimicrobial peptides [28]. AMPs are typically composed of cationic and hydrophobic amino acids, comprising a total length of 12–50 residues and they are generally amphipathic. Notably, not only the presence of cationic and hydrophobic residues but also their distribution in the peptide sequence can influence both antimicrobial activity and cytotoxicity [29]. Even though the precise mechanism of action of AMPs is still unknown, they are believed to permeabilize the bacterial membrane, leading to no specificity in targeting, hence less chance of development of antimicrobial resistance [30]. Positively charged AMPs interact with the negatively charged bacterial membrane, via electrostatic forces. In several proposed models, AMPs either bind [31], insert themselves into the membrane [32] or cover the membrane in a detergent-like manner [33]. However, studies have also demonstrated that some AMPs have intracellular targets such as inhibition of transcription, translation, or other processes (e.g., DNA, RNA, guanosine tetraphosphate (ppGpp) and protein synthesis), or can interfere with bacterial cell wall synthesis [34]. AMPs have been widely investigated as antimicrobial agents, especially against planktonic bacteria. Within those studies, AMPs have shown reduced development of resistance. Though resistance to these molecules seems to be less common than to conventional antibiotics, it was recently found in several pathogens during the co-evolution of host and pathogens [35]. A number of AMPs are currently undergoing clinical and pre-clinical trials [36]. During those trials, a number of candidates failed due to high susceptibility to enzymatic degradation and fast renal clearance leading to a short peptide in vivo half-life or unexpected in vivo toxicity. Apart from the mentioned drawbacks, AMPs inherently possess other disadvantages such as low bioavailability, pH- and/or salinity-dependent activity, and loss of activity due to binding to serum proteins [33,37].

As a solution, rational design including synthetic optimization of the active peptide, such as altering length, backbone, side chains, hydrophobicity, charge and amphipathicity can be used to overcome limitations, yielding in peptide mimics (also called peptidomimetics) [33,36]. The resulting mimics usually retain the activity profile of the original AMP, while displaying significant improvement in bioavailability and overall stability. Within recent years, development of peptidomimetics has allowed for manipulation of the pharmacological profile of AMPs, resulting in a deeper understanding of the relationship between structure and antimicrobial activity. Additionally, many AMPs have also been shown to modulate the immune responses in the host, like interfering with unwanted inflammation and recruit immune cells to the site of infection [26]. Thus, AMPs and their mimics are considered to have a great potential for local delivery from implant coatings.

This review discusses recent developments in the field of antibiofilm peptides and peptidomimetics as well as presents an overview of their applications for coating the surface of medical implants, as well as methods for their immobilization.

## 2. Antibiofilm Peptides

In recent years, a number of excellent reviews have debated the antibiofilm properties of both natural and synthetic AMPs, and for an in-depth analysis, we refer the readers to these reviews [38,39,40,41]. In this current paper we will only briefly highlight some key peptides and mimetics which have been studied in vitro and in vivo and paved the way for different surface coating strategies.

### 2.1. Antibiofilm Peptides Tested In Vitro

Within the past decade, a number of peptides have demonstrated excellent antibiofilm activity leading to either inhibition or eradication of the biofilm. The selected examples of such peptides are presented in Table 1. One of the first peptides that was discovered to possess antibiofilm activity was the human cathelicidin LL-37 [42]. LL-37 is one of the most studied AMPs, displaying antibiofilm activity at sub-inhibitory concentrations (i.e., 16-fold below its minimum inhibitory concentration (MIC) value against planktonic *P. aeruginosa*) [43]. Notably, this peptide has virtually no activity against planktonic bacteria and yet display a biofilm-specific activity. This discovery has triggered the interest to investigate antibiofilm properties of synthetic fragments of LL-37 in order to understand the relationships between peptide’s structural properties and its mode of action [44]. In one particular study, a peptide library screen for small cationic peptides, derived from LL-37, resulted in identification of a nine-mer antibiofilm peptide 1037 (KRFRIRVRV-NH_2_) that inhibited *P. aeruginosa* biofilm formation by 50% at one-twentieth the MIC [45]. LL-37 analogs were also tested against *Staphylococcus aureus*, where the most active analog P10 demonstrated remarkable activity inhibiting biofilm development and potentially eradicating a preformed biofilm of multi-drug resistant clinical isolate of *S. aureus* [46]. 

Similarly, the mouse homologue cathelicidin-related antimicrobial peptide(CRAMP) has been able to inhibit fungal biofilm formation [47]. More interestingly, several studies have demonstrated that shorter fragments of CRAMP, such as AS10, still were able to inhibit biofilm growth of *Candida albicans*, while showing no cytotoxicity [48]. Additionally, to antifungal activity, AS10 exhibited inhibitory activity against bacterial biofilms formed by *Escherichia coli* and *P. aeruginosa*. Human liver-derived antimicrobial peptide hepcidin 20 (hep20) serves as another example of natural peptide with antibiofilm properties. When tested against *Staphylococcus epidermidis* biofilms, hep20 was able to inhibit biofilm formation [49]. These findings spiked development of various antibiofilm synthetic peptides derived from natural AMPs. For example, innate defense regulator peptide IDR-1018 was derived from bovine neutrophil host defense peptide bactenecin and demonstrated broad-spectrum antibiofilm activity against a number of both Gram-positive and Gram-negative pathogens [50]. As a next step, de la Fuente-Núñez et al. designed and screened a d-enantiomeric peptide library based on properties associated with IDR-1018 [51]. Resulting two synthetic peptides DJK-5 and DJK-6 displayed broad-spectrum antibiofilm activity and ability to eradicate pre-existing biofilms. Mataraci and Dosler used a different approach and designed a hybrid peptide named CAMA (cecropin (1-7)–melittin A (2-9) amide) that contained the N-terminal region from cecropin A and N-terminal region from melittin A and was highly effective at inhibiting methicillin-resistant *Staphylococcus aureus* (MRSA) biofilm formation [52]. Anunthawan et al. presented two synthetic tryptophan-rich cationic antimicrobial peptides KT2 and RT2 that demonstrated potency against multidrug-resistant *E. coli* biofilms at sub-MIC levels. Both peptides were able to prevent biofilm formation and eradicate mature biofilms [53]. In a recent study, Al-Balas and colleagues demonstrated a synthetic ultrashort peptide UP-5 containing only arginine and biphenylalanine that showed potency against *S. aureus* biofilms [54]. Other interesting examples are described in detail by de la Fuente-Núñez et al. and di Luca et al. [39,55].

AMPs are known to successfully enhance potency of conventional antibiotics especially against multi-drug resistant strains [56,57]. Combinational therapy reduces antibiotic concentrations and hence, lowers toxicity and resistance development. Similar approach was utilized in order to combat biofilm-related infections since those are highly difficult to eradicate using antibiotics alone [58]. This matter has been fully described by de la Fuente-Núñez et al., who concluded that synthetic peptides can successfully potentiate ineffective antibiotics to treat biofilms [39]. For example, earlier mentioned, IDR-1018 exhibited synergistic interactions with all of the conventional antibiotics when tested against biofilms formed by at least one of the six tested bacterial species (i.e., *P. aeruginosa*, *E. coli, Acinetobacter baumannii*, *Klebsiella pneumoniae*, MRSA and *Salmonella enterica*) [59]. Another example of combining cationic peptides and conventional antibiotics was demonstrated by Mataraci and Dosler [52]. The authors explored the combinations of different classes of antibiotics with indolicidin, CAMA, and nisin against *S. aureus* biofilms, where synergistic effect was found in the majority of the cases.

### 2.2. Structure-Activity Relationship Studies of Antibiofilm Peptides

In the wake of increasing attention to AMPs as antibiofilm agents, Batoni and colleagues developed a database dedicated to AMPs active against biofilms [60]. To date this database provides information about 221 peptides accompanied by target organisms, experimental methods, peptide concentration and percentage of biofilm inhibition/reduction. However, only several studies attempted to understand the link between the structure of peptides and their antibiofilm activity. De la Fuente-Núñez et al., designed several peptide libraries of small cationic peptides and screened them for antibiofilm activity [45,61]. First, when incorporating d-amino acids no obvious correlation was found between enantiomeric composition and antibiofilm activity. Secondly, no relationship between peptide activity against planktonic bacteria and biofilm was observed. Embracing a similar approach, Hancock and colleagues tackled antibiofilm testing of peptides using computer-aided discovery [62]. In their study, a peptide library consisting of 96 single amino acid substituted variants of IDR-1018 was synthesized and tested against *S. aureus* biofilms. Collected data were used to establish quantitative structure-activity relationship (QSAR) models relating the antibiofilm activity of tested peptides to hundreds of molecular descriptors derived from their sequences. The developed 3D-QSAR models then predicted the probability that a peptide would possess antibiofilm activity from a library of 100,000 virtual peptide sequences in silico. A subset of these variants was SPOT-synthesized and their activity assessed, revealing that the QSAR models resulted in ~85% prediction accuracy. In another study, Gnoatto and colleagues explored the antibiofilm properties of peptides from a structure-activity relationship perspective by analyzing sequences of 85 peptides with Gram-positive and Gram-negative antibiofilm activity [63]. In their findings, presence of such amino acids as lysine (K), arginine (R), leucine (L), valine (V) and phenylalanine (F) was crucial for the antibiofilm activity of peptides. Generally, active peptides contained from 12 to 20 amino acids, thus length of peptide also appeared to be of importance. In order to achieve potency against biofilm formed by Gram-negative bacteria, the length of peptide was suggested to be of 20 amino acids, with a frequency up to 7 K, 5 R, 3 alanine (A), 2 V, 2 F and 2 L. By contrast to efficacy against Gram-positive bacterial biofilms, 17 amino acids were enough, where presence of such amino acids as alanine, lysine and arginine were of crucial importance. Apart from length and charge properties, authors were not able to establish any correlation between secondary structure and biofilm activity. Overall, more studies are required in order to identify key peptide structural features responsible for biofilm prevention and eradication.

### 2.3. Mechanism of Action

Though AMPs play a crucial role in the innate immune system of virtually all living organisms, their mechanism of action towards both planktonic bacteria and biofilms are poorly investigated. In their reviews, Batoni et al., and Di Luca et al., explored possible mechanisms of interactions that take place between peptides and biofilms [38,55]. Peptides can be divided in two groups according to the concentration required to inhibit or reduce/eradicate biofilms. The first classical group contains peptides that are active at concentrations equal or higher than MIC against correspondent planktonic bacteria. These peptides are thought to inhibit biofilms by killing either planktonic biofilm forming bacteria, cells detaching from biofilm or biofilm embedded bacteria. However, more interestingly, peptides that display specific antibiofilm potency at concentrations much lower than MIC fall into a second group with a non-classical mechanism of action. For example, Overhage et al., demonstrated that LL-37 was able to inhibit biofilm formation at 16-fold lower concentration compared to its MIC through possible dysregulation of genes involved in the biofilm formation and/or mortility [43]. Notably, another antibiofilm peptide HBD3 appeared to interfere with expression of genes responsible for the synthesis of the major extracellular polysaccharide PIA (polysaccharide-intercellular-adhesin) [64]. By contrast, peptide hep-20 was proved to reduce the biomass of growing biofilms of both PIA-positive and PIA-negative bacterial strains of *S. epidermidis* via interfering with extracellular matrix accumulation without affecting cell viability [49]. While maintaining no effect on planktonic cells, peptide IDR-1018 has shown wide range of antibiofilm activity against both Gram-positive and Gram-negative bacteria at low concentrations. It is thought to be involved in a direct interaction with intracellular nucleotide ppGpp that coordinates the stringent response [61,65]. In a parallel study, two d-enantiomeric peptides DJK-5 and DJK-6 were suggested to inhibit biofilm formation and suppress mature biofilms via targeting the intracellular nucleotides guanosine pentaphosphate (p)ppGpp, using similar route as first suggested for IDR-1018 [51]. Both classical and non-classical mechanisms of the antibiofilm activity of AMPs are presented in detail by Batoni and colleagues [38].

### 2.4. Antibiofilm Peptides Tested In Vivo

Despite a high interest in AMPs with antibiofilm properties, there have been only a few studies investigating their activity using in vivo models (Table 2). This may be explained by the lack of efficient animal models. To date, very few animal models are available that involve biofilm-associated infections and they are often inconsistent, technically challenging and self-limiting, frequently resolving without antibiotic treatment [66].

One of the drawbacks of antimicrobial peptides includes their enzymatic degradation. To overcome this limitation, several groups designed full d-enantiomeric peptides, which cannot be recognized by bacterial or host proteases that can cleave peptides composed entirely of l-amino acids [67]. In their study, Mohamed et al. investigated potency of a d-enantiomeric peptide D-RR4 alone and in combination with colistin against *P. aeruginosa* and *A. baumannii* biofilms in vivo using the *C. elegans* infection model [68]. D-RR4 produced a significant reduction in bacterial burden of *P. aeruginosa* at 8 × MIC (85%). Similarly, colistin (8 × MIC) produced an 89% reduction in bacterial load. Complete clearance of bacteria from worms was achieved only when D-RR4 was combined with colistin. In case of colistin-resistant *P. aeruginosa* isolated from a cystic fibrosis patient, treatment with D-RR4 resulted in 75% reduction. Combination of D-RR4 and colistin showed enhanced killing reducing 98% of colistin-resistant *P. aeruginosa* in infected worms. Similar patterns were observed for *A. baumannii* biofilm treatment. Additionally, D-RR4 significantly increased the survival rate of *Caenorhabditis elegans* and gave nearly complete protection after 24 h for both pathogens. It is worth mentioning, that both the *C. elegans* model and the *Galleria* model are often used as established biofilm infection models [69,70]. Another study also exploring the effect of d-enantiomer peptides tested DJK-5 and DJK-6 in both models in order to explore their activity against *P. aeruginosa* biofilms [51]. Peptides IDR-1018 and RI-1018 (retro-inverse d-analog of IDR-1018) were also included in the study, however only RI-1018 managed to display protective effect in *Galleria mellonella*. On the other hand, DJK-5 and DJK-6 significantly protected both *C. elegans* and *G. mellonella* from *P. aeruginosa* infections. Another peptide SAAP-148, formulated in ointment, effectively treated both acute and established (biofilm-associated) wound infections in mice caused by multi-drug resistant *S. aureus* and *A. baumannii* after a single 4 h treatment [71]. WRL3 also demonstrated antibiofilm activity against multi-drug resistant *S. aureus* infection using a MRSA-infected burn wound model [72]. WRL3 was found to be able to control proliferation of MRSA in wound tissue as well as reduce bioburden and provided a more favorable environment for wound healing. Recently, Hancock et al. have developed a reproducible mouse model of cutaneous abscess infections and used it for testing IDR-1018 alongside with a new peptide 3002 [62]. After a single intra-abscess injection, the lesion size significantly reduced by 2.8-fold (for 3002) and 2.4-fold (for IDR-1018). Interestingly, neither peptide had any effect on the bacterial load recovered from the abscesses suggesting that the decrease in abscess size may be due to antibiofilm activity combined with additional immunomodulatory effects, induced by the peptides. Additionally, Hancock and his colleagues used the same in vivo model to demonstrate that both DJK-5 and DJK-6 peptides were able to treat cutaneous abscesses caused by both PAO1 strain and a clinical strain of *P. aeruginosa* by decreasing skin lesion sizes and reduced bacteria load inside the abscess [65].

It is important to underline difficulties in testing antibiofilm properties of peptides in both in vitro and in vivo settings that may render comparison among various peptides. Batoni et al. discusses in detail various factors that can influence the testing results, such as lack of a standardized operating protocols resulting in utilizing different methods and models for assessment of the peptide antibiofilm activity [38]. Azeredo et al., have also reviewed an existing nowadays lack of consensus among the diversity of techniques used to grow and study biofilms [73]. Additionally, they emphasized difference in the biofilm testing by comparing all known biofilm models. Therefore, more studies should aim to address those issues in order to obtain comparable data.

## 3. Antibiofilm Peptidomimetics

When straddling towards the post antibiotic era, AMPs were viewed as the solution. However, antimicrobial peptides possess a number of drawbacks such as susceptibility to protein degradation, salt/pH sensitivity etc. Peptidomimetics, on the other hand, have proved to overcome stability issues while retaining activity profile of original peptides. However, despite their advantages, there are only a few peptidomimetics in the literature tested for antibiofilm potency.

One of the first mimetics to be reported on in respect to anti-biofilm properties was a mimetic containing poly-*N*-substituted glycine residues, or a peptoid. In contrast to peptides, the peptoids are substituted at the amide nitrogen and cannot form intermolecular hydrogen bonds to stabilize secondary structures [74]. The 12-mer peptoid sequence, Peptoid 1, composed of alternating Nlys (*N*-(4-aminobutyl) glycine) and Nspe (*N*-(1-phenylethyl) glycine) units (Table 3) and a lipidated analogue of it, Peptoid 1-C13_4mer_, were analyzed in a stationary biofilm assay with *P. aeruginosa.* At MICs concentrations of Peptoid 1 and Peptoid 1-C13_4mer_, resulted in a significant reduction in bacterial biomass and cell viability of the biofilm [75]. In other words, both Peptoid 1 and Peptoid 1-C13_4mer_ killed bacterial cells within biofilm. The authors propose that Peptoid 1 oligomerization via aromatic side chain interactions promotes and increased local Peptoid 1 concentration at the cell membrane, thus effectively disrupting the bacterial biofilm. Conversely, Peptoid 1-C13_4mer_ is suggested to disrupt the hydrophobic exopolysaccharide and penetrate deeper into the matrix as a result on the lipid tail, thus efficiently reducing cell viability. A similar effect of lipidation has been demonstrated in a family of lipo-cyclic-γ-AApeptides (Table 3), resulting in comparable or enhanced performance compared to ciprofloxacin in the prevention of biofilm formation for both Gram-positive and Gram-negative bacteria [76]. The most potent peptidomimetic in this family, YL-36, exhibited up to 80% and 70% reduction in the formation of the *P. aeruginosa* and MRSE (methicillin resistant *Staphylococcus epidermidis*) biofilms. Combined with earlier discussed results, lipidated antimicrobial peptidomimetics are suggested to be more effective in the biofilm prevention, possibly due to the existence of lipid tails, which might retard the growth of biofilms.

Another well-studied peptidomimetic approach has been to make peptide–peptoid hybrids. Using this strategy and alternating lysine units and Nspe units, resulted in a sequence with similarities to Peptoid 1, though with a higher charge to hydrophobicity ratio. The hybrid HDM-4 (Table 3) exhibited strong anti-biofilm activity at the same sub-MIC level concentration toward four separate Gram-negative bacteria, as also concluded for cationic anti-biofilm peptides [77]. For all tested bacterial strains, the biomass was reduced by >90% at 0.125–0.53 μg/mL planktonic cell MIC. A similar peptide–peptoid hybrid has been designed with the use of β-peptoid elements rather than Nspe, thus introducing more flexibility along the backbone and a more elongated structure. Testing this β-peptoid-peptide hybrid 4d (Table 3) demonstrated that it was able to reduce cell viability by 80–85% at 1–4 × MIC [78]. However, 4d managed to cause 78% of reduction in living biomass only at 400 × MIC concentration. Interestingly, in the other study a similar alternating scaffold was utilized where Nspe residues were replaced with *N*-alkyl/aryl pyrazole (Py) residues and lysine was substituted with arginine to maintain the necessary cationicity. One of the most potent examples of pyrazole-derived peptidomimetics is ultra-short peptidomimetic Py11 that was able to inhibit 90% biofilm development of multidrug-resistant *P. aeruginosa* at 16 µg/mL compared to LL-37 that reached the same result at 64 µg/mL [79]. Olygo-acyl lysyl antimicrobial peptides (OAK), oligomers of acetylated lysines with alternating pattern of hydrophobic residues (amino acyl chains) and cationic amino acids similar to previously mentioned peptidomimetics, have also shown not only antimicrobial but also antibiofilm activity. The most active representative was lapidated OAK that displayed potency in killing preformed biofilm of *Streptococcus mutans*, however only at significantly higher concentrations (e.g., ≥0.5 mM) within one hour of exposure [80] (Table 3).

As a different approach, several peptidomimetics were designed with an alternative backbone. For example, a short amphiphilic cationic peptidomimetic 23b contained-amino-[1,10-biphenyl]-3-carboxylic acid backbone with biphenyl attached with hydrophobic Trp and cationic aminoethyl guanidine [81]. Such peptidomimetic was able to disrupt biofilms of *S. aureus* and *E. coli* (41% and 39% respectively) at 250 µM (Table 3). These findings may indicate that the increased cationic charge and hydrophobicity potentiates the activity against planktonic cells, however does not facilitate disruption of the large aggregates of bacteria surrounded by an extracellular matrix. Other ultra-short dipeptidomimetics designed using spermine backbone, inhibited MRSA biofilm formation at sub-MIC concentrations. Though both showed better killing efficacy than vancomycin, 1c and 1d were able to kill viable cells of preformed biofilms only at concentrations higher that MIC, indicating no antibiofilm specificity [82]. Further modifications led to the discovery of dialkyl cationic amphiphile peptidomimetics bearing two identical length lipophilic alkyl chains and one non-peptidic amide bond, still keeping the previously established hydrophobicity–cationicity ratio. The most potent dialkyl cationic amphiphile 4g was not able to show high potency while inhibited 80% of the *S. aureus* and *E. coli* biofilms at 18–50 × MIC, while eradicated preformed biofilms at values corresponding to 64-fold of MIC values [83].

Though mentioned peptidomimetics have displayed some antibiofilm activity, it remains far from desirable. Overall, more investigations are needed in this field in order to understand how to enhance antibiofilm profile of peptidomimetics.

## 4. Surface-Immobilized Peptides on Medical Implants

**Figure 1:**
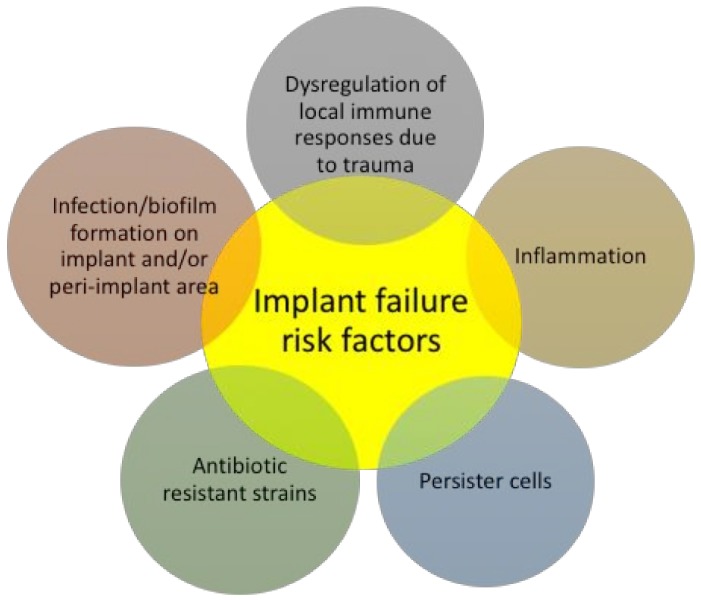
Medical implant failure risk factors.

Medical implant failure or malfunctioning are complex phenomena and can be attributed to many factors (Figure 1). To start with, the surface of medical implants can be colonized by bacteria during the implantation process, or at a later time point [14,15], putting the performance and longevity of the implant at risk. Moreover, due to peri-implant tissue damage during the implantation procedure, the local immune responses at the implant-tissue interphase are dysregulated, thus rendering the peri-implant area more vulnerable to infections. These factors render the treatment of biomaterial-associated infection very complex.

Traditional treatments, including prophylactic and systemic administration of antibiotics, or even combinations of them, often are unsuccessful in combating infections on medical implants, because of failing in establishing a sufficient antibiotic concentration at the implant site [20,21,22]. Hence, localized delivery of antimicrobial agents potentially eliminates issues such as systemic toxicity and excessive exposure to the antimicrobial agent, favouring the rising of resistant bacteria populations, and is therefore highly desirable [15,23,24,25]. The development of resistance is a major problem for currently available antibiotic coatings, with one study indicating that gentamicin-resistant *Staphylococci* were recovered from gentamicin-loaded beads implanted in a patient after arthroplasty [84], highlighting the dangers of the development of resistance from exposure to biomaterial-associated antimicrobials. Another important factor for the longevity of medical implants in vivo is the reduction of the inflammation at the peri-implant area (Figure 1) [85,86], usually caused by bacterial components, such as lipopolysaccharide (LPS), a component of the Gram-negative bacteria cell wall. LPS-induced macrophage recruitment at the implant site plays a key role in implant acceptance, as it initiates the inflammatory response, by pro-inflammatory cytokine release [85,86]. However, few studies evaluate the potential of inflammatory reactions induced by implant coating [87]. On top of their antimicrobial activity, some AMPs also display anti-inflammatory properties by inhibiting the secretion of pro-inflammatory cytokines and chemokines, such as tumor necrosis factor (TNF)-α [88] and upregulating the secretion of anti-inflammatory cytokines, such as IL-10 [89].

**Figure 2:**
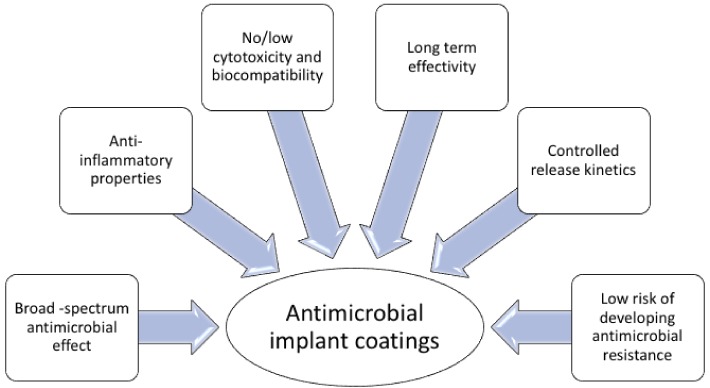
Requirements for antimicrobial coatings on medical implants.

A promising antimicrobial strategy for the prevention of bacterial colonization on such surfaces and localised delivery of antimicrobial drugs at the peri-implant area, is coating the surface of the implant with antimicrobial compounds. There are two key questions one should raise when designing biomaterial with surface immobilized AMPs. For one, immobilization of peptide on a surface will potentially help to keep the surface sterile. But, for how long should the peptide be available on the surface? Should it be degraded or released? In order to achieve an ideal antimicrobial coating, for localized delivery of antimicrobial compound, it should display the following properties (Figure 2): (i) broad-spectrum antimicrobial activity; (ii) controlled kinetics of release and effective concentration when released, exceeding the MIC [90]; (iii) long term efficacy, resistance to degradation in vivo; (iv) biocompatibility; (v) stability under heat-sterilization; (vi) limited capacity to induce antimicrobial resistance. Moreover, depending on the use and the purpose, medical implants can be considered permanent e.g., orthopedic implants, artificial heart valves, or can be used on a temporary basis e.g., catheters, sutures, contact lenses. Due to this diversity, various types of materials are required for the manufacturing of medical implants, rendering the development of antimicrobial coatings more perplexing.

Conventional antibiotics, such as vancomycin and gentamicin, have been incorporated in antimicrobial coatings for orthopedic implants [91], however they failed to fulfil all of the above-mentioned criteria. The main drawbacks of coatings are the antibiotics limited antimicrobial activity and the release of drugs at levels below the reported MIC, that may evoke bacterial resistance [92,93]. On the other hand, it is more difficult for bacteria to develop resistance against AMPs, and for example repeated exposure to sub-inhibitory concentrations of melimine (TLISWIKNKRKQRPRVSRRRRRRGGRRRR), a synthetic peptide produced by combining the active portions of mellitin and protamine, did not induce resistance [94]. Moreover, high doses of antibiotics often harm cell viability and osteogenic activity and may impair osteointegration, which is a vital process for the acceptance of orthopedic implants [95].

Several biomaterials have been functionalised with AMPs, including titanium [96,97,98,99], catheter-like surfaces [100,101,102,103] and contact lenses [94]. A challenge for all AMP incorporation in coatings is that the immobilization process on an implant surface should not prohibit the antibacterial activity of the AMP, i.e., the diffusion of the peptides into the bacterial membrane. It has been reported that immobilized antimicrobial drugs often lose their activity, as they do not reach their cellular targets [104]. In addition, altered stereochemistry or orientation of the peptide molecule resulted in lower antimicrobial efficacy compared to when in solution [101,102]; though increased antibacterial properties have also been reported as a result of tethering AMPs to a surface [105]. Moreover, the concentration of the AMP on the surface as well as its concentration when released, should be appropriate, in order to exert antimicrobial effect. All these parameters have to be considered when developing a new AMP coating.

There is a plethora of methods for coating biomaterials, broadly categorized as methods for peptide immobilization, and peptide release from a biomaterial surface. Peptide immobilization methods usually include sophisticated chemical modifications, as producing biomaterial coatings by means of physical adsorption or electrostatic attraction on the surface of the biomaterial is usually unsuitable, due to instability. Otherwise, such coatings will be weak and instable mechanically, during the invasive processes of implant insertion, and at the in vivo biological environment with the biological fluid flow. Thus, a stronger platform is required for the bonding between the AMP and the coating, for example, by means of covalent bonding. Covalent immobilisation would allow for no or very low leakage of the antimicrobial agent at the peri-implant area, allowing for a limited antibacterial effect in the surrounding area. Moreover, a number of biomaterials, such as titanium, have inert surfaces, thus, the surface of such an implant has to be modified to acquire additional properties, such as antimicrobial, bone formation-promoting or other [106]. As a result, harsh chemical conditions and complicated, multiple steps are required—including, for example, coupling with silane agents. On the other hand, for other applications like food packaging coatings, surgical instruments, where release of the antimicrobial agent is undesired, covalent immobilizations offer several advantages by providing stable bonds between the bioactive compound and the functionalised surface [104]. Another strategy offers active release coatings, where the antimicrobial compound coated on the implant is not covalently bound, therefore is released, taking care of peri-implant infections, which often occur due to the dysregulated immune system around the implants [107,108,109,110,111,112].

There is a great variability in how different studies assess the potential of antimicrobial biomaterial coatings, in terms of in situ antimicrobial activity on the implant and at the peri-implant area, in vitro and in vivo, biocompatibility, etc. The majority of studies reported solely in vitro assays, with only a few in vivo studies [97,98,113,114,115,116,117]. The methodology for evaluating the in vitro antibacterial and anti-biofilm activity is not unified among the literature, with studies testing the bacterial load directly by measuring the colony forming units, others by performing live-dead staining or fluorescent based staining methods (Table 4). Critically, most of the in vitro work reviewed in the present article, in order to assess the effects of the antimicrobial coatings on the targeted bacteria, used static or mild agitation culture conditions. Those culture conditions do not simulate the conditions of biofilm formation and growth in vivo, in a human body, therefore the results must be interpreted accordingly. For example, the constant flow of biological fluids in the human body applies significant shear forces and supplies sustained nutrition to the bacteria. These conditions impact the bacterial growth and biofilm formation; therefore, it is of high importance to take them into account. Sawant and colleagues showed that the formation and growth of *E. coli* biofilm on surfaces in a drip flow bioreactor was twice that obtained in a shaker [118]. This indicates that biofilm grown in the drip flow bioreactor system mimics the in vivo conditions better and presents a greater challenge to the antimicrobial peptide coatings than standard culture conditions. A few studies have used flow systems for studying the antibacterial effect of biomaterial coatings, with the example of Chen and colleagues, who used a drip flow biofilm reactor system to show the antimicrobial activity of covalently bound GL13K peptide on titanium discs against *Streptococcus gordonii* biofilm [119].

Titanium and titanium alloys confer excellent mechanical properties and biocompatibility, due to the formation of stable oxide layer on its surface [120]; therefore, titanium is widely used for the manufacturing of orthopedic and dental implants. In the following sections different immobilization strategies of AMPs on titanium implants are explored.

### 4.1. Antimicrobial Peptides Immobilised on Titanium

**Figure 3:**
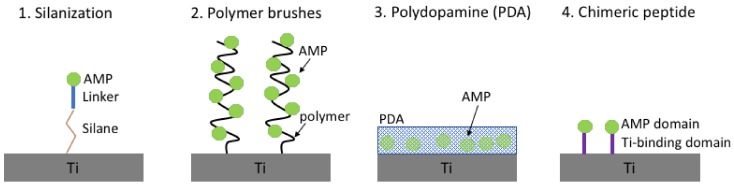
Covalent immobilisation of AMPs on titanium surfaces.

Silanization (Figure 3) is a low-cost and effective covalent coating method used to modify material surfaces that are rich in hydroxyl groups by introducing silane active groups (e.g., amino group and carboxyl group), which can easily be modified by further grafting. The first study, to the best of our knowledge, which explored the immobilization of the cathelicidin related AMP LL-37 on titanium via silanization was carried out by Gabriel and colleagues [121]. Interestingly, they showed that a flexible and long spacer containing polyethylenoglycol (PEG) chains is required between the silane and the AMP, in order to maintain its antibacterial effect against *E. coli* [121]. In this study, immobilized LL-37 alone had a significantly lower antimicrobial effect compared to when it was coupled to the flexible spacer chains. Moreover, the use of PEG as spacer additionally led to intrinsic anti-adhesive properties of the treated surface [122].

Silanization has been successfully used for the immobilization of AMPs on titanium in a number of studies (Table 4). The human lactoferrin-derived peptide hLf1-11 anchored to titanium surfaces via silanization exhibited an outstanding reduction in the adhesion and early stages of biofilm formation of the oral cavity pathogens *Streptococcus sanguinis* and *Lactobacillus salivarius* [123]. Similarly, when using silanization to immobilize the synthetic, broad-spectrum antimicrobial peptide melimine on titanium surfaces, the coating significantly reduced the in vitro adhesion and biofilm formation of *P. aeruginosa* and *S. aureus* on the surfaces compared to the non-modified titanium [96]. Notably, it was further demonstrated that the melimine-coated titanium reduced bacterial colonization in vivo, in both mouse and rat subcutaneous infection models and that the coated implants retained activity even after ethylene oxide gas sterilization [96].

As grafting of the AMPs away from the surface using different spacer elements has demonstrated positive impact on activity, there were attempts to develop this concept further. Thus, polymer brushes (Figure 3) have attracted considerable attention as a way to modify the surface properties of materials [136], and in comparison to other surface modification methods (e.g., silanization), polymer brushes have increased density of functional groups on their surface, allowing the conjugation of a higher number of biomolecules, i.e., AMPs [125]. Among techniques used to prepare polymer brushes, the “grafting from” approach based on surface-initiated atom transfer radical polymerization offers an experimentally straightforward route to graft polymer brushes on biomaterial, in a controlled and reproducible manner [137]. Polymer brushes, apart from servicing as scaffolds for the attachment of other molecules, like AMPs, exert non-adhesive properties, a characteristic highly desirable for biomaterial. The AMP Tet-20 (Table 4) conjugated to polyacrylamide brushes grafted on titanium surfaces, retained its antimicrobial properties against *P. aeruginosa* both in vitro and in vivo, in a rat model [98]. Furthermore, increased peptide densities were achieved on the brush layer, compared to direct grafting of peptides on the material surface [97].

For clinical applications, it would be ideal to achieve the surface modification of titanium implants by simple procedures; for example, spraying a drug-loaded solution onto the implant immediately before the surgery, which can form a stable coating layer that is strongly bound to the implant in situ. The methods discussed so far (silanization, polymer brushes) involve harsh chemical conditions and are more cumbersome. A simpler approach, using a dopamine coating as a scaffold for the loading of AMPs, has been investigated in several studies [113,114,128,138]. Inspired by the mussel’s strong adhesion to virtually any wet surface, attributed to a unique protein with special amino acid composition [139], Messersmith and colleagues reported a method to form thin multifunctional polymer coatings through simple dip-coating of objects in an aqueous solution of dopamine, as a mimic of the mussel’s adhesive protein [140]. The mussel’s strong adhesive properties are believed to be related to the overexpression of an amino acid bearing a catechol group (l-3,4-dihydroxyphenylalanine, DOPA) and lysine residues that are in close proximity with the substrate interface [141]. Dopamine combines the chemical properties of both DOPA and lysine, hence, it has been chosen as an analogue of the mussel’s adhesive protein. The dopamine-modified surfaces were shown to be potent substrates for further ad-layer deposition of various compounds [140,142,143]. The reactivity of the dopamine-coated surfaces relates to the formation of poly-di-hydroxy-indole (polydopamine, PDA) as a thin layer which under high pH conditions, oxidizes to the corresponding poly-ortho-quinone-indole. The catechol side chains of DOPA are believed to form strong charge-transfer complexes to metal oxide surfaces, such as titanium [144,145]. The resulting film can subsequently interact covalently with various compounds via Schiff-base reactions (amine containing molecules) or Michael type reactions (amine and thiol containing molecules) [140,143]. PDA coating is attractive for medical implant coating manufacturing, due to its high efficiency of surface conjugation and its convenient property of naturally coating of unmodified substrates.

Bacitracin immobilised on a PDA scaffold on titanium retained its antimicrobial activity against Gram-positive bacteria, and furthermore, it was found that this coating enhanced osteogenic differentiation of human bone marrow mesenchymal stem cells and reduced the inflammation both in vitro and in vivo in a rat model [113,138]. PDA layers have also been used for dual coating of titanium, with both AMP and another molecule, to gain dual functionality. A dual coated titanium with the AMP SESB2V and vascular endothelial growth factor retained the antimicrobial properties of SESBV2 (Table 4) against *Bactillus cereus* and *E. coli* [128] and enhanced fibroblast proliferation. Furthermore, these dual coated implants were tested in vivo in a rabbit keratitis model where they prevented peri-implant bacterial infection, compared to raw titanium implants [114].

Another alternative to the conventional titanium chemical immobilization methods is a bio-friendly immobilization strategy utilizing chimeric peptides, comprised of an AMP domain and a specific titanium-binding domain, usually with a flexible linker in between. These bifunctional chimeric peptides are able to bind selectively to the titanium surfaces, while exposing the AMP domain. Among other peptides able to recognise the surface of inorganic material [146], peptide TBP-1 has been isolated by a phage system for its ability to bind on titanium surfaces via electrostatic interactions [147]. Mutational analyses revealed that the N-terminal motif RKLPDA (named minTBP-1) is responsible for binding, with the arginine, proline and aspartate residues being the most important. It has been proposed that the electrostatic interactions take place between the charged residues of minTBP-1 and the charged surface of titanium [148] under physiological conditions [144], which is supported by atomic force microscopy data [149]. Chimeric peptides comprised of titanium-binding domain and AMP connected by a linker have been successfully immobilised on titanium surfaces and retained their antimicrobial activity [127,129]. In order to maintain the functionalities of both titanium binding and antimicrobial activity, it was shown that a rigid linker is preferable compared to a flexible one [127].

Recombinant silk proteins are considered a promising biomaterial due to their self-assembly properties, cell biocompatibility and low immunogenicity. Nilebäck and colleagues used silk proteins, recombinantly fused to the antimicrobial peptide Magainin I [150]. The silk fusion protein can self-assemble, without covalent attachment, on a variety of biomaterial including titanium, hydroxyapatite, stainless steel, and polystyrene [150]. Silk coatings demonstrated improved antimicrobial potency, and stability toward washing with hydrochloric acid, sodium hydroxide, and ethanol.

### 4.2. Antimicrobial Peptides Released from Titanium

**Figure 4:**
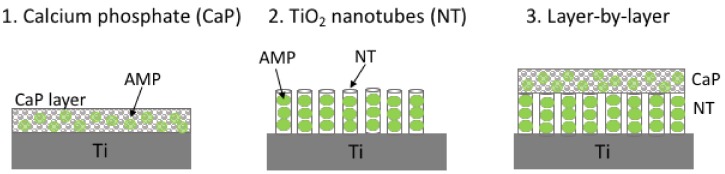
AMP release coating strategies on titanium surfaces. CaP: calcium phosphate coating; NT: TiO_2_ nanotures.

Coatings aimed to release antimicrobial compounds are often considered preferable for orthopedic implants due to release of the antimicrobial agent at the peri-implant area. However, a suitable drug-loading system has to be developed that can effectively deliver a sustained amount of antimicrobials on the surface of implants, without impairing peri-implant bone growth. That means a depot of the antimicrobial molecule has to be stored on the surface of the implant and released in a controlled manner over a preferentially prolonged period. Among several reported systems that store antimicrobial agents on the implant, calcium phosphate (CaP) coatings, titanium dioxide (TiO_2_) nanotubes, layer-by-layer assembly and hydrogels are discussed below (Figure 4).

CaP is a porous ceramic which can be coated on titanium implants, as it improves the integration of the implant to the bone and enhances bone growth [151,152]. Thus, the combination of CaP and an antimicrobial peptide has potential to promote osteointegration of the implant and provide antimicrobial protection at the same time. AMPs can be successfully loaded to the CaP-coated titanium substrates through a simple soaking method, that has been reported for loading Tet213 (Table 4) [131]. This implant had broad-spectrum antimicrobial activity in vitro against both Gram-positive and Gram-negative pathogens, and retained its potency after repeated uses, while remaining biocompatible to osteoblast-like cells. Another technique for the delivery of AMPs is the formation of TiO_2_ nanotubes on the surface of titanium implants. These nanotubes can significantly accelerate osteoblast growth and adhesion in vitro [153] and improve bone formation and bonding strength in vivo [154,155,156]. Even though the design of TiO_2_ nanotubes was reported in other studies [157,158,159,160,161], it was Ma and colleagues, who first tested loading self-organized, vertically oriented TiO_2_ nanotubes as local delivery system of AMPs, using HHC-36 [162]. As a result, peptide loaded TiO_2_ nanotube surface effectively killed *S. aureus* in vitro, and the release of the AMP from the nanotubes was verified via liquid chromatography coupled mass spectrometry (LC-MS) analysis [134]. Furthermore, GL13K peptide loaded in TiO_2_ nanotubes, on titanium surfaces, by a simple soaking technique demonstrated a sustained and slow drug release profile in vitro and eradicated the growth of *Fusarium nucleatum* and *P. gingivalis* within 5 days of exposure [133].

Due to the fact that both CaP coating and TiO_2_ nanotubes adsorb peptide, the release rate in both systems is fairly rapid, with previous studies suggesting a maximum cumulative release amount only within a few hours [131]. Therefore, these AMP delivery methods are unsuitable for long-term peri-implant infection, where sustained release is required for a longer period. Hence, in order to prolong the drug release, layer-by-layer (LBL) antimicrobial coatings on titanium implants have been studied, using both CaP and vertically aligned TiO_2_ nanotubes, both loaded with HHC-36 (Table 4) [132]. These films were topped with a thin palmitoyl-oleoyl phosphatidyl-choline phospholipid (POPC) film to control the release of the peptide [163,164]. POPC is found naturally in eukaryotic cell membranes, has antimicrobial properties, and also is suitable for bone cell attachment [165] and osteointegration in vivo [166].

Other LBL strategies, involve electrostatic interactions between charged polyanions and polycations, in a number of layers, producing a film. Each layer entraps antimicrobial agent, and through degradation of each layer, continuous release is achieved over time. Titanium surface coating using an LBL assembly involving Tet213, chitosan and hyalouronic acid demonstrated excellent and sustained long-term antimicrobial activity against *S. aureus* and *P. gingivalis* [117]. Moreover, the surface promoted the cellular attachment, accompanied by low levels of cytotoxicity and erythrocyte haemolysis.

Another technology developed for the sustained release of antimicrobial from coatings for 3–4 weeks includes multi-alternating layers of polymer and lipid biodegradable coating, called the Polymer Lipid Encapsulation Matrix (PLEX), containing polylactic-co-glycolic acid, dipalmitoyl phosphatidyl choline and distearoyl phosphatidyl choline [167,168]. The PLEX was used in combination with OP-145 to coat intramedullary nails, which were then placed in rabbit bone, and resulted in improved clearance of *S. aureus* infection, compared to uncoated implants, both on the implant and at the peri-implant tissue [115]. Moreover, the PLEX method has been recently used to capture two AMPs at the same time, SAAP-145 and SAAP-276, both derivatives of LL-37, with antimicrobial and anti-biofilm properties. When SAAP-145 and SAAP-276 were incorporated in a PLEX coating, a constant release was achieved for up to 30 days, after an initial burst release of >50% of the loaded peptides. In a murine model for biomaterial-associated infection, SAAP-145-PLEX and SAAP-276-PLEX coatings significantly reduced the number of culture positive implants, in an *S. aureus* infection model [116].

In situ formation of the coating on the implant is facile for clinical applications, for example via spraying the antimicrobial matrix on the implant in the surgical room. A gelatin-based hydrogel, functionalised with catechol motifs to promote its binding efficiency on titanium and loaded with HHC-36 has been reported, and, to our knowledge, it is the only study in which the AMP coating is applied on the implant via spraying [130]. A burst release of the AMP was observed, with a cumulative release of 37% within the first 24 h after incubation and a relatively steady release for the next 20 days of incubation, resulting in a final cumulative release to roughly 90%.

### 4.3. Other Materials

Polyethylene is a biomaterial used in joint replacement, and like titanium, it is inert. Surface oxidation approach is usually used to create functional groups suitable for further chemical reaction. PEG was used as a hydrophilic spacer molecule, linking the polyethylene surface and E14LKK peptide (LKKLLKLLKKLLKL), derived from magainin, which was attached to the free terminus of PEG. The PEG spacer additionally provided mobility to the tethered peptides. This modification demonstrated antimicrobial activity in broth cultures against *E. coli* [169]. Additionally, polymyxin B retains its microbial activity against *E. coli*, when covalently bound to glass surface, via a silane coating [170]. Studies of several coatings for catheter material were carried out, but yielded either low biocompatibility, or inefficient long-term/limited antimicrobial activity [171]. So far, AMP coatings on catheters have only been tested in one in vivo study [172], where peptide E6 (RRWRIVVIRVRR-NH_2_) was attached to polyurethane material of catheter, using a polymer tethering approach. The peptide-coated catheter pieces were shown to have antibacterial properties against both Gram-positive and Gram-negative bacteria, in vitro in both culture medium and urine. In addition, they were shown to be effective in a mouse in vivo urinary trach infection model. The synthetic peptide melimine, was found to reduce *P. aeruginosa* and *S. aureus* adhesion to contact lenses both when adsorbed or when covalently attached, and furthermore melimine was resistant to heat sterilization [94].

Recently, a synthetic, tryptophan-rich peptide tethered on polyethylene terephthalate was capable of preventing *S. aureus* biofilm formation in vitro, while not being toxic to human cell lines [173]. In another study, plant-derived, cyclic AMPs coupled to steel surfaces via polydopamine, demonstrated antibacterial and antibiofilm activity against *S. aureus* [174]. Chitosan films have also been used as platforms for the covalent immobilization of AMP due to the intrinsic osteogenic and antibacterial properties of chitosan [175,176,177,178]. Covalent immobilization of the human lactofericin peptide hLF1-11 on chitosan films, both with and without a PEG spacer was designed. However, no improvement in the antibacterial properties was observed, as the functionalization with hLF1-11 without a PEG spacer increased the bacterial adhesion to chitosan films [179], though the viability of attached bacteria was decreased, especially with the PEG spacer.

## 5. Surface-Immobilized Peptidomimetics

Several non-natural mimics of antimicrobial peptides with high activity have recently been developed, providing advantages in terms of chemical diversity and significant resistance to protease degradation [180]. There is a virtually unlimited compositional versatility obtained through variation of side chain composition, with both natural and non-natural side chains [181].

As peptidomimetics offer several advantages over peptides, including resistance to protease degradation, they could naturally have a potential application as biomaterial coatings; however, the work done so far is limited. The main studies exploring the potential of peptidomimetics as coatings for biomaterials mostly include work on peptoids [182,183]. In 2005, Messersmith’s group reported for the first time a new class of antifouling chimeric polymers, consisting of a peptoid and a peptide domain for robust adsorption on biomaterial surface, inspired by the previously mentioned mussel’s adhesive protein (DOPA-lysine oligopeptide) [184]. The chimeric peptoid with methoxyethyl side chains that mimicked PEG chains was successfully adsorbed on titanium by simple immersion of the titanium in an aqueous solution of the polymer [185]. Modified titanium surfaces showed a remarkable protein resistance profile, exhibiting a 100-fold reduction in adsorbed protein, when exposed to human serum compared to unmodified titanium. Moreover, peptoid-modified titanium surfaces exhibited low levels of fibroblast attachment for over 5 months under the constant presence of fresh serum. However, no bacterial experiments were performed in order to investigate the fouling of the modified surface by bacteria in this initial study. In 2008, the same group showed that the attachment of both *E. coli* and *S. epidermidis* was significantly reduced on the peptoid-modified titanium surfaces, compared to unmodified, for up to four days under continuous-flow conditions [186]. Moreover, methoxyethyl side chains provided superior long-term fouling resistance compared to hydroxyethyl and hydroxypropyl side chains [186]. Additionally, a 20-mer peptoid, coupled with a DOPA-Lysine penta-peptoid via a spacer and immobilised on titanium surfaces, compromised the membranes of attached bacteria [187]. Later on, it was demonstrated that poly *N*-methyl glycine (poly-sarcosine) brush coatings resist mouse fibroblast attachment over an extended period of 7 weeks, while being challenged with repeated seeding with fresh cells, while resisting attachment of both *E. coli* and *P. aeruginosa* [188].

Based on the recent reports of zwitterionic anti-fouling surfaces [189,190], the addition of zwitterionic elements to a polymer brush already possessing antifouling properties was investigated [191]. The peptoid polymers consisted of equimolar analogues of glutamic acid and lysine, separated by a different number of *N*-substituted methoxyethyl glycine as spacer; however, these did not show improved potency in resisting bacterial cell attachment compared to the control brushes without the zwitterionic elements [191].

Poly(dimethylsiloxane) (PDMS)— and poly(ethylene oxide) (PEO)— based block copolymer coatings functionalized with amphiphilic, surface-active, and sequence-controlled oligomer side chains were tested in biofouling assays using the macroalga *Ulva linza*. It was found that the presence of the side chains of the peptoid facilitated the removal of sporelings from the PDMS block copolymer. Both the initial attachment and adhesion strength of the diatom *Navicula incerta* were lower on the coatings based on PEO compared to those based on PDMS [192].

## 6. Conclusions

Biofilm-associated infections are difficult to treat, since biofilms are difficult to kill or eradicate, while they are contributing to surface persistent contamination and the prevalence of nosocomial pathogens. Importantly, none of the existing antibacterial treatments have been developed specifically against biofilms, yet biofilms account for roughly 80% of all infections, both chronic and surface-associated. Biomaterial-associated infections remain a major constraint to the long-term use of medical implants and impose a great economic burden to healthcare providers, especially under the prism of the steadily increasing use of medical implants in the aging population. Developing alternative strategies for the treatment of both chronic and implant-associated infections is of paramount importance for the longevity and the safe use in medicine. Despite recent progresses, improvement of an antimicrobial delivery system with antibiofilm activity, avoiding resistance development and cytotoxic side effects is still a challenge.

AMPs emerge as promising tools in the war against biofilm-related infections. Though AMPs spiked a high interest as they display high potency against biofilms, limitations like biodegradation are often flagged. However, it is important to realize that biodegradation of AMPs in vivo, predominately have been investigated for solubilized peptides, thus the stability and half-life of tethered AMPs might be significantly less damaging for the application of AMPs as antimicrobial coatings. Another bottleneck for clinical use of AMPs is the lack of mode-of-action understanding, or the dirty drug remark, pointing towards the AMPs’ multiple mechanisms of action. Not surprisingly, tethering of AMPs might change their mode-of-action, but this change might actually be used, as covalent linking of the peptide simply might cause the peptides to target the cell membrane.

To overcome drawbacks related to protease degradation, peptidomimetics seem as a logical evolvement. Though performing very well in the antimicrobial field, peptidomimetics strive to show potency against biofilms. Therefore, more studies with more diverse representation of peptidomimetics are needed. Even though peptidomimetics offer several advantages over AMPs, including protease resistance, their potential as biomaterial coating antimicrobial agents has only been poorly explored so far.

Regardless of the choice of AMPs or peptidomimetics, when preparing coated surfaces, the device specifications and usage area will influence the immobilization strategy, thus adding a second layer of complexity. Lastly, investigations of the antibiofilm properties of the surface can benefit from employment of similar structure activity relationship techniques that drove optimization of classical AMPs back at the beginning of this century. In parallel to computational solutions, it is crucial to establish a more uniform protocol for antibiofilm measurements.

## Figures and Tables

**Table 1:** Examples of antibiofilm antimicrobial peptides (AMPs) tested in vitro.

AMP	Sequence	Bacteria	Activity	Ref
LL-37	LLGDFFRKSKEKIGKEFKRIVQRIKDFLRNLVPRTES	*P. aeruginosa*	Inhibition	[43]
AS10	KLKKIAQKIKNFFQKLVP	*C. albicans*	Inhibition	[48]
		*P. aeruginosa*		
		*E. coli*		
IDR-1018	VRLIVAVRIWRR-NH_2_	*P. aeruginosa*	Inhibition/Eradication	[50]
		*E. coli*		
		*A. baumannii*		
		*K. pneumoniae*		
		*S. enterica*		
		*MRSA*		
DJK-5	vqwrairvrvir **	*P. aeruginosa* *E. coli* *A. baumannii* *K. pneumonia* *S. enterica*	Inhibition/Eradication	[51]
DJK-6	vqwrrirvwvir **			[51]
KT2	NGVQPKYKWWKWWKKWW-NH_2_	*E. coli*	Inhibition/Eradication	[53]
RT2	NGVQPKYRWWRWWRRWW-NH_2_			[53]
CAMA	KWKLFKKIGIGKFLQSAKKF-NH_2_	MRSA	Inhibition	[52]
P10	LAREYKKIVEKLKRWLRQVLRTLR	MDR *S. aureus*	Inhibition/Eradication	[46]
UP-5	RBRBR *	MRSA	Inhibition	[54]
hep20	ICIFCCGCCHRSHCGMCCKT	*S. epidermidis*	Inhibition	[49]

* B-biphenylalanine; ** lower case letters represent d-amino acids. Full species names are: *Pseudomonas aeruginosa*, *Candida albicans*, *Escherichia coli*, *Acinetobacter baumannii*, *Klebsiella pneumoniae*, *Salmonella enterica*, MRSA: methicillin-resistant *Staphylococcus aureus*, MDR: multidrug resistance *S. aureus*, *Staphylococcus epidermidis*.

**Table 2:** Examples of antibiofilm AMPs tested in vivo.

AMP	Sequence	Bacteria	In Vivo Model	Ref
SAAP-148	LKRVWKRVFKLLKRYWRQLKKPVR	*A. baumannii*	A mouse wound skin model	[71]
		MRSA		
3002	ILVRWIRWRIQW-NH_2_	MRSA	A mouse cutaneous abscess model	[62]
IDR-1018	VRLIVAVRIWRR-NH_2_			
DJK-5	vqwrairvrvir *	*P. aeruginosa*	*Caenorhabditis elegans* nematodes *Galleria mellonella* larvae A mouse cutaneous abscess model	[51,65]
DJK-6	vqwrrirvwvir *			
WRL3	WLRAFRRLVRRLARGLRRNH_2_	MRSA	An infected burn mouse wound model	[72]
D-RR4	wlrrikawlrrika-NH_2_ *	*P. aeruginosa*	*C. elegans* model	[68]
		A. baumannii		

* Lower case letters represent d-amino acids.

**Table 3:** Examples of peptidomimetics with antibiofilm properties.

Compound	Structure	Bacteria	Activity	Ref.
1	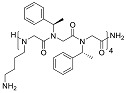	*P. aeruginosa*	Inhibiotion/Prevention	[75]
1-C13_4mer_	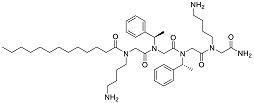	*P. aeruginosa*	Inhibition/Prevention	[75]
Y-36	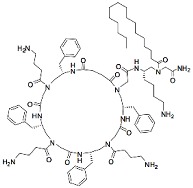	*P. aeruginosa*	Inhibition/Prevention	[76]
		*MRSE*		
HDM-4	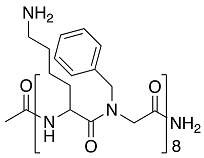	*E. coli*	Inhibition	[77]
		*P. aeruginosa*		
		*S. enterica* Typhimurium		
		*A. baumannii*		
		*K. pneumoniae*		
4d	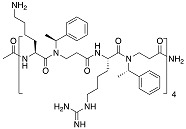	*S. epidermidis*	Inhibition	[78]
Py11	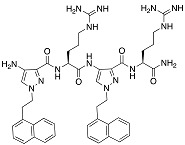	*P. aeruginosa*	Inhibition	[79]
C_14_KKc_12_K	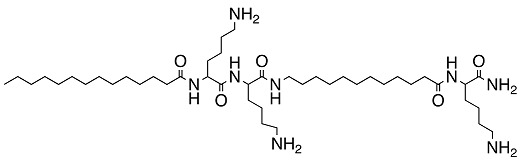	*Streptococcus mutans*	Inhibition	[80]
23b	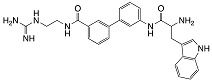	*S. aureus*	Inhibition	[81]
		*E. coli*		
4g	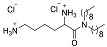	*S. aureus*	Inhibition	[83]
		*E. coli*		
1c, 1d	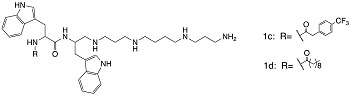	*S. aureus*	Inhibition	[82]

**Table 4:** Examples of AMPs immobilized on titanium with different strategies.

Biomaterial	AMP	Sequence	Coating Method	Bacteria	In Vitro Testing	In Vivo Testing	Biocompatibility Tested on	Ref.
Immobilised								
Titanium, disks or hollow round casings	Melimine	CTLISWIKNKRKQRPRVSRRRRRRGGRRRR	Three-step: Silanization with APTES ^1^ through vapor deposition. Cross linker addition sulfo-SMCC ^2^ by immersion. Cys-peptide addition by immersion.	*S. aureus* strain 38 *P. aeruginosa* PAO1	Bacterial adhesion via fluorescence microscopy	Mouse and rat subcutaneous infection models. CFU determination.	n/a	[96]
Titanium, commercially pure Grade II discs	GL13K	GKIIKLKASLKLL-NH_2_	Two-step: Silanization with (3-chloropropyl)triethoxysilane Peptide addition on the silane by immersion.	*Streptococcus gordonii* strain ML-5	Drip Flow Bioreactor Culture CFU assay, ATP Assay, L/D staining BacLight, SEM	n/a	n/a	[119]
Titanium	GZ3.163	4-methylhexanoyl-Cys-d-Dab-Dab-Dab-Leu-d-Phe-Dab-Dab-Leu-NH_2_	Three-step: Silanization with APTES ^1^. PEGylation with NHS-PEG_24_-MAL ^3^ ester, by immersion. Peptide coating by immersion.	*E. coli* DH5α *P. aeruginosa* ATCC 27853 *S. aureus* ^10^	CFU assay, L/D staining BacLight, SEM	n/a	Mouse blood cells lysis assay	[124]
Titanium, platelets	LL-37	CLLGDFFRKSKEKIGKEFKRIVQRIKDFLRNLVPRTES	Three-step: Silanization with APTES ^1^. PEG linker: NHS-PEG-Mal ^4^. Incubation with peptide.	*E. coli* strain K12	Bacterial killing assay (Propidium iodide staining)	n/a	n/a	[121]
Titanium, deposited on silicon wafer	Tet213	KRWWKWWRRC	Three-step: Copolymer brushes ^5^ synthesized on Ti. Modification of the grafted chains using 3-maleimidopropionic acid *N*-hydroxysuccinimide ester Peptide conjugation via cysteine residue.	*P. aeruginosa* PA01 (*luxCDABE*)	CFU assay, luminescence	n/a	n/a	[97]
Titanium, deposited on silicon wafer	Tet-20	KRWRIRVRVIRKC	Silanization with APTES ^1^. *N*-substituted polyacrilamide brushes. One-end tethering of AMP.	*P. aeruginosa* PA01 (*luxCDABE*)	CFU assay, luminescence, SEM	Rat subcutaneous infection model	MG-63 human osteoblast-like cells, Platelet activation, Complement activation analysis	[98]
Titanium, commercially pure Grade II	hLF1-11	MPA-Ahx-Ahx-Ahx-GRRRRSVQWCA-NH_2_^6^	Three-step: Silanization with APTES ^1^. Bifunctional cross-linker iodoacetic acid *N*-hydroxysuccini- mide ester Peptide addition, by immersion.	*S. sanguinis* ^10^ *L. salivarius* ^10^	CFU assay, L/D staining BackLight, CLSM, BacTiter-Glo Reagent for biofilm	n/a	Human foreskin fibroblasts	[125]
Titanium, commercially pure Grade II	hLF1-11	MPA-Ahx-Ahx-Ahx-GRRRRSVQWCA-NH_2_^6^	Three-step: Silanization with either APTES ^1^ or CPTES ^7^. Addition of the bifunctional cross linker 3-(maleimide)propionic acid *N*-hydroxysuccinimide ester. Peptide addition, by immersion.	*S. sanguinis* ^11^ *L. salivarius * ^11^	CFU assay, SEM, luminescence BacTiter-Glo Reagent for biofilm	n/a	Human foreskin fibroblasts	[123]
Titanium, commercially pure Grade II	GL13K	GKIIKLKASLKLL-NH_2_	Two-step: Silanization with CPTES ^7^. Peptide addition by immersion.	*Porphyromonas gingivalis* ATCC 33277	ATP assay, CFU assay	n/a	Human gingival fibroblasts (HGF) and MC3T3-E1 murine osteoblasts	[126]
Titanium foils 99.2% pure	Ti-binding- linker-JPH8194	RKLPDA-PAPAP-KRLFRRWQWRMKKY	Chimeric peptide, with titanium-binding domain.	*S. gordonii* ATCC 51656, *S. sanguis* ATCC 10556	L/D staining BacLight, CLSM	n/a	MC3T3-E1 Osteoblasts Culture	[127]
Titanium alloy, Ti6AL4V	Bacitracin	Ile-Cys-Leu-d-Glu-Ile-cy(Lys-d-Orn-Ile-d-Phe-His-d-Asp-Asp)	Polydopamine	*S. aureus* ATCC 25923, MRSA	n/a	Rat model, Ti rods were implanted into the femurs. CFU on the implant and at the peri-implant tissues.	Histopathology evaluation of the bone tissue around the Ti rod implant. nephrotoxicity of bacitracin-modified Ti in vivo	[113]
Titanium	SESB2V	[(RGRKVVRR)_2_K]_2_KK	Polydopamine	*S. aureus* ATCC 29213 *P. aeruginosa* ATCC 9027	L/D staining BacLight	rabbit keratitis model, CFU/cornea	n/a	[114]
Titanium alloy, Ti6Al4V	SESB2V	[(RGRKVVRR)_2_K]_2_KK	Polydopamine	*B. cereus* ATCC 14579 *E. coli* ATCC 35218	L/D staining, fluorescent microscopy	n/a	Human corneal stroma cells from donors tissue	[128]
Titanium, grade V powder	AMP1	LKLLKKLLKLLKKL	Chimeric peptide, with titanium-binding domain.	*E. coli* ATCC 2592 *S. mutans* ATCC 25175 *S. epidermidis* ATCC 29886	SYTO9 green fluorescent nucleic acid stain fluorescent microscopy	n/a	n/a	[129]
	AMP2	KWKRWWWWR						
**Release**								
Titanium	HHC-36	KRWWKWWRR-NH_2_	hydrogel, cathehol functionalised, addition of AMP	*P. aeruginosa* *E. coli* *S. aureus* *S. epidermidis*	CFU assay, SEM	n/a	human mesenchymal stem cells	[130]
Titanium	OP-145	Ac-IGKEFKRIVERIKRFLRELVRPLR-NH_2_	PLEX ^8^ coating, mixed with peptide. Immersion for in vitro testing, spraying for in vivo.	*S. aureus* clinical strain JAR060131	CFU assay, Crystal violet	Mouse subcutaneous and Rabbit intramedullary nail infection models. Biopsy fom skin, subcutaneous tissue and implant.	n/a	[115]
Titanium	Tet213	KRWWKWWRRC	Calcium phosphate by electrolytic deposition, soaking in the AMP solution.	*P. aeruginosa* H1001: *lux-CDABE* *S. aureus* ATCC 25293	CFU assay, luminescence	n/a	MG-63 human osteoblast-like cells	[131]
Titanium	HHC-36	KRWWKWWRR-NH_2_	LBL ^9^ coating. Three layers of vertically oriented TiO_2_ nanotubes, a thin layer of calcium phosphate coating and a phospholipid.	*P. aeruginosa* H1001: *lux-CDABE* *S. aureus* ATCC 25293	CFU assay, SEM	n/a	MG-63 human osteoblast-like cells Platelet activation Red blood cell (RBC) haemolysis assay	[132]
Titanium	GL13K	GKIIKLKASLKLL-NH_2_	TiO_2_ nanotubes.	*F. nucleatum* ATCC 25586 *P. gingivalis* ATCC 33277	CFU assay	n/a	MC3T3-E1 cells, a clonal mouse preosteoblastic cell line, J774A.1 mouse macrophage	[133]
Titanium	HHC36	KRWWKWWRR	TiO_2_ nanotubes, adsorption via a simple vacuum-assisted physical adsorption method.	*S. aureus* ATCC 25293	CFU assay, SEM	n/a	n/a	[134]
Titanium alloy, Ti6Al4V	Cateslytin	RSMRLSFRARGYGFR	Hydrogel made of natural polysaccharide, sodium alginate, modified by catechol groups along the polymer chain.	*P. gingivalis* ATCC 33277	Alamar Blue cell viability assay CFU assay	n/a	Gingival fibroblasts HGF-1	[135]
Titanium, solid medical grade implants	SAAP-145	Ac-LKRLYKRLAKLIKRLYRYLKKPVR-NH_2_	Biodegradable PLEX was mixed with peptide.	*S. aureus* JAR060131, MDR *S. aureus* LUH15101	Propidium iodine fluorescence	mouse model of subcutaneous biomaterial-associated infection. CFU on the implant and at the peri-implant area. Biopsies.	n/a	[116]
Titanium plaHNUtes	Tet213	KRWWKWWRRC	layer-by-layer assembly, chitosan, hyalouronic acid. AMP was covalently linked to free amines of collagen IV	*S. aureus* ATCC 25923, *P. gingivalis* ATCC 33277	CFU and fluorescent microscopy	mice, intraperitoneal administration	Cytotoxicity Assay. HaCaT cells Human erythrocytes, haemolysis assay In vivo immunotoxicity assay.	[117]

^1^ APTES: 3-Aminopropyl triethoxysilane; ^2^ Sulfo-SMCC: 4-(*N*-maleimidomethyl)cyclohexane-1-carboxylic 3-sulfo-*N*-hydroxysuccinimide ester; ^3^ NHS-PEG_24_-MAL: succinimidyl-[*N*-maleimidopropionamido]-poly(ethylene glycol); ^4^ NHS-PEG-Mal: α-*N*-hydroxysuccinimidyl-ω-maleimidyl-PEG; ^5^ Copolymer brushes: *N*,*N*-dimethylacrylamide-*co*-*N*-(3-aminopropyl)-methacrylamide hydrochloride); ^6^ Ahx: 6-aminohexanoic acid, as spacer, MPA: 3-mercaptopropionic acid, as anchoring group; ^7^ CPTES: (3-chloropropyl)triethoxysilane; ^8^ PLEX: biodegradable Polymer-Lipid Encapsulation MatriX, consisting of poly lactic-*co*-glycolic acid, dipalmitoyl phosphatidyl choline, distearoyl phosphatidyl choline and cholesterol; ^9^ LBL: Layer-by-Layer; ^10^ Strains not specified; ^11^ *Streptococcus sanguinis* (CECT 480, Colección Española de Cultivos Tipo (CECT), Spain) and *Lactobacillus salivarius* (CCUG 17826, Culture Collection University of Göteborg (CCUG), Sweden). n/a: not applicable, CFU: Colony Forming Units, L/D staining: Live/Dead staining, SEM: Scanning Electron Microscopy, CLSM: Confocal laser scanning microscopy.
